# Restoration of Cathepsin D Level via L-Serine Attenuates PPA-Induced Lysosomal Dysfunction in Neuronal Cells

**DOI:** 10.3390/ijms231810613

**Published:** 2022-09-13

**Authors:** Hyunbum Jeon, Yeo Jin Kim, Su-Kyeong Hwang, Jinsoo Seo, Ji Young Mun

**Affiliations:** 1 Neural Circuit Research Group, Korea Brain Research Institute, Daegu 41062, Korea; 2 Department of Brain Sciences, Daegu Gyeongbuk Institute of Science and Technology (DGIST), Daegu 42988, Korea; 3 Department of Pediatrics, School of Medicine, Kyungpook National University, Daegu 41944, Korea; 4 Astrogen Inc., 440, Hyeoksin-daero, Dong-gu, Daegu 41072, Korea

**Keywords:** L-serine, lysosome, lipid droplet, propionic acid (PPA), neuron

## Abstract

L-serine is a non-essential amino acid endogenously produced by astrocytes and is abundant in human diets. Beneficial roles of the metabolic products from L-serine in various conditions in the brain including neuronal development have been reported. Through several preclinical studies, L-serine treatment was also shown to offer beneficial therapeutic effects for brain damage such as ischemic stroke, amyotrophic lateral sclerosis, and Parkinson’s disease. Despite evidence for the value of L-serine in the clinic, however, its beneficial effects on the propionic acid (PPA)-induced neuronal toxicity and underlying mechanisms of L-serine-mediated neuroprotection are unknown. In this study, we observed that PPA-induced acidic stress induces abnormal lipid accumulation and functional defects in lysosomes of hippocampal neurons. L-serine treatment was able to rescue the structure and function of lysosomes in PPA-treated hippocampal neuronal cells. We further identified that L-serine suppressed the formation of lipid droplets and abnormal lipid membrane accumulations inside the lysosomes in PPA-treated hippocampal neuronal cells. Taken together, these findings indicate that L-serine can be utilized as a neuroprotective agent for the functionality of lysosomes through restoration of cathepsin D in disease conditions.

## 1. Introduction

L-serine is an amino acid endogenously produced by astrocytes and is abundant in human diets [1]. L-serine has been found to be a valuable therapeutic agent in vitro/in vivo studies and human clinical trials [2]. D-serine, however, has limited clinical use because of its potential nephrotoxicity [3] and poor diffusion through the blood–brain barrier [4]. Beneficial effects of L-serine in various pathological conditions have been reported. It was shown to promote dendritic elongation and branching of Purkinje neurons [5]. As a precursor of various nucleotides including L-cysteine, phosphatidyl-L-serine, and sphingolipids, L-serine is required for the neuronal development process [6]. Because L-serine is also a precursor of neurotransmitters including glycine and D-serine, it is a major player in metabolic pathways related to synaptic activity and plasticity [7]. L-serine has been considered as a potential drug for ischemic stroke, having shown neuroprotective effects when administered at an early stage after cerebral ischemia in rats [8], improving the recovery of neurological functions, such as learning, memory, sensory, motor, and balance functions in the rat model for stroke [9]. L-serine is also considered as a potential therapeutic for neurodegenerative diseases. It has also shown a safe effect in phase I clinical trials in amyotrophic lateral sclerosis patients [10]. Dietary supplementation with L-serine restored various deficits in an Alzheimer’s disease mouse model [11]. Despite increasing evidence for the value of L-serine in neuroprotection, further studies to elucidate the mechanism of action are needed.

In order to investigate how L-serine protects neurons, we utilized hippocampal neuronal cells treated with propionic acid (PPA) as an in vitro model of neurotoxicity in this study. PPA is a short-chain fatty acid (SCFA) that an essential role in the human body, including increasing enteric smooth muscle contractions [12,13] and the stimulation of host defense peptide expression [14]. However, excess levels of PPA cause propionic acidemia (PA), which has been associated with brain atrophy, dementia, cognitive disorders, and motor impairment [15,16]. PPA can penetrate the blood–brain barrier to modulate energy metabolism, neurotransmitter synthesis and release, and lipid metabolism when the concentration of SCFAs in the gut is increased [17]. Our previous results showed that increased Peroxisome proliferator-activated receptor gamma coactivator-1 alpha (PGC1a), a regulator of fatty acid metabolism, and decreased Acyl-CoA synthetase-1 (ACSL1) facilitate long-chain fatty acid transport in SH-SY5Y neuronal cells [18]. A mixture of SCFAs increased carnitine palmitoyltransferase 1 (CPT1), which affects fatty acid oxidation, and decreased the levels of CD36, the fatty acid translocase in a pig model [19]. PPA produced defects in autophagy with a decrease in the number of autolysosomes, suggesting the existence of a degradation defect [20]. The lysosome is a key organelle regulating lipid metabolism [21]. In this study, we tested the neuroprotective effects of L-serine in a PPA-treated in vitro model. To precisely examine whether and how L-serine regulates PPA-induced lysosomal alterations, we employed multiple imaging tools including confocal microscopy, correlative light and electron microscopy (CLEM), and transmission electron microscopy (TEM). When L-serine was co-administered with PPA to hippocampal neuronal cells, PPA-induced formation of lipid droplets and their colocalization with lysosomes was significantly suppressed. Biochemical analysis showed that the levels of lysosomal aspartic protease and cathepsin D (CTSD) were significantly changed in hippocampal neuronal cells by PPA, which were ameliorated by L-serine. Taken together, these data showed that L-serine possess protective potentials against toxin-induced functional and structural changes of lysosomes by resolving lysosomal lipid accumulation in hippocampal neurons.

## 2. Results

### 2.1. Toxicological Effects of Propionic Acid and L-Serine on HT22 Cells

**Figure 1:**
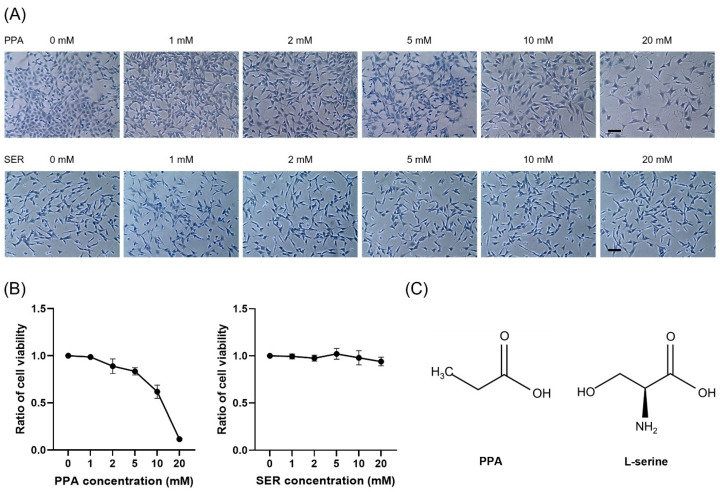
The effect of different concentrations of propionic acid (PPA) or L-serine (SER) on the viability of HT22 cells. (**A**) Representative images of HT22 cells stained with trypan blue. Scale bar, 100 μm. (**B**) Six PPA concentrations from 0 to 20 mM in DMEM medium were set. HT22 cells were incubated in a medium with different PPA concentrations for 48 h. Cell viability was measured by CCK-8 assay (*n* = 3 replicates). Cells were treated with equal amounts of L-serine (SER) or PPA in DMEM medium for 48 h. L-serine (SER) has no toxicity in HT22 cells. (**C**) Chemical structures of PPA and L-serine.

To investigate the effect of PPA on the viability of HT22 hippocampal neuronal cells, we treated the cells with different doses of PPA. We determined the concentration of PPA that substantially changed cell viability. Cell viability decreased rapidly following treatment with 10 mM PPA (Figure 1A). This result indicated that a concentration of 5 mM PPA was not toxic to HT22 cells, and this concentration was used in further investigations of the effect of PPA on the structure of cell organelles in HT22 cells. L-serine (SER) itself did not affect the viability of the cells (Figure 1B).

### 2.2. PPA Decreased Lysosomal Activity in PPA-Treated HT22 Cells, and L-Serine Attenuated the Dysfunction

**Figure 2:**
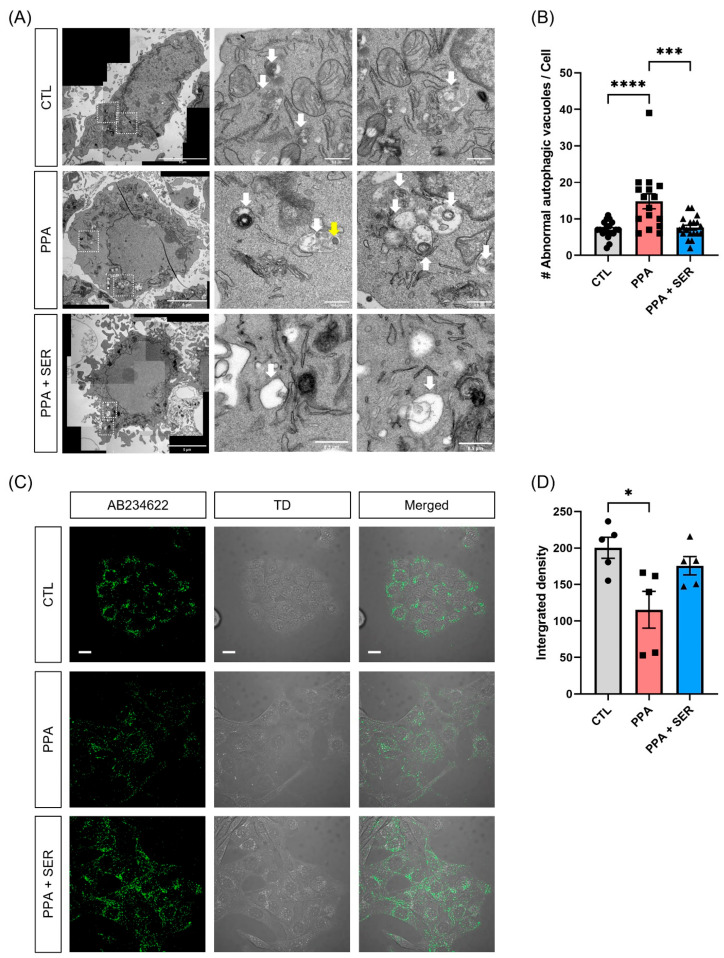
PPA induced lysosomal abnormality and decreased lysosomal activity in HT22 cells. Cells were treated with 5 mM PPA only or 5 mM PPA and 10 mM L-serine (SER) for 48 h. (**A**) Representative electron microscopic images of autolysosome in HT22 cells. PPA induced the lipid membrane-bounded inside the lysosomes in HT22 cells. The white arrow represents the autolysosome structure, and yellow arrow represents the lipid droplet inside the autolysosome. Scale bar, 5 μm (left); 0.5 μm (the magnified images). (**B**) Quantification of the abnormal autophagic vacuoles analyzed by TEM images (*n* = 15). SEM, *** *p* < 0.001, **** *p* < 0.0001 by one-way ANOVA, Tukey’s multiple-comparison test. (**C**) Representative fluorescence images of the release of the self-quenched substrate in HT22 cells. Confocal images showed lysosomal activity with green fluorescence. Scale bar, 20 μm. (**D**) Quantification of the integrated density of lysosomal activity in HT22 cells (*n* = 5). PPA significantly reduced lysosomal activity compared to control. L-serine (SER) restored lysosomal activity compared to PPA. * *p* < 0.05 by one-way ANOVA, Tukey’s multiple-comparison test. TD: transmitted light detector image. AB234622: Lysosomal intracellular activity assay kit.

We observed abnormal autophagic vacuoles (lysosomes) and accumulated lipid membranes as well as the accumulation of lipid droplets (LD) inside the lysosomes in PPA-treated HT22 cells using TEM (Figure 2A). The structural changes observed in the lysosomes were similar to those produced by treatment with the well-known lysosomal inhibitor, bafilomycin A1 [22]. As a result of TEM analysis, the number of abnormal autophagic vacuoles (lysosomes) including lipid accumulations significantly increased in HT22 cells treated with only PPA. However, cells co-treated with PPA and L-serine showed a significantly decreased number of the abnormal autophagic vacuoles (Figure 2B). We hypothesized that PPA affects lysosomal activity as a result of lysosomal dysfunction and induces the accumulation of lipid membranes. To investigate whether PPA affected the lysosomal activity, we performed an intracellular lysosomal activity assay in HT22 cells treated with PPA or co-treated with PPA and L-serine. We analyzed lysosomal activity using confocal microscopy. Green puncta represent the lysosomal activity (Figure 2C). As shown in Figure 2D, HT22 cells treated only with PPA showed significantly decreased lysosomal activity compared with the control group. However, cells co-treated with PPA and L-serine had increased lysosomal activity compared with PPA-only treated cells. These results showed that PPA induced lysosomal dysfunction, but L-serine increased lysosomal activity in HT22 cells.

### 2.3. PPA Increased Lipid Droplets in HT22 Cells, and L-Serine Attenuated the Lipid Accumulation

**Figure 3:**
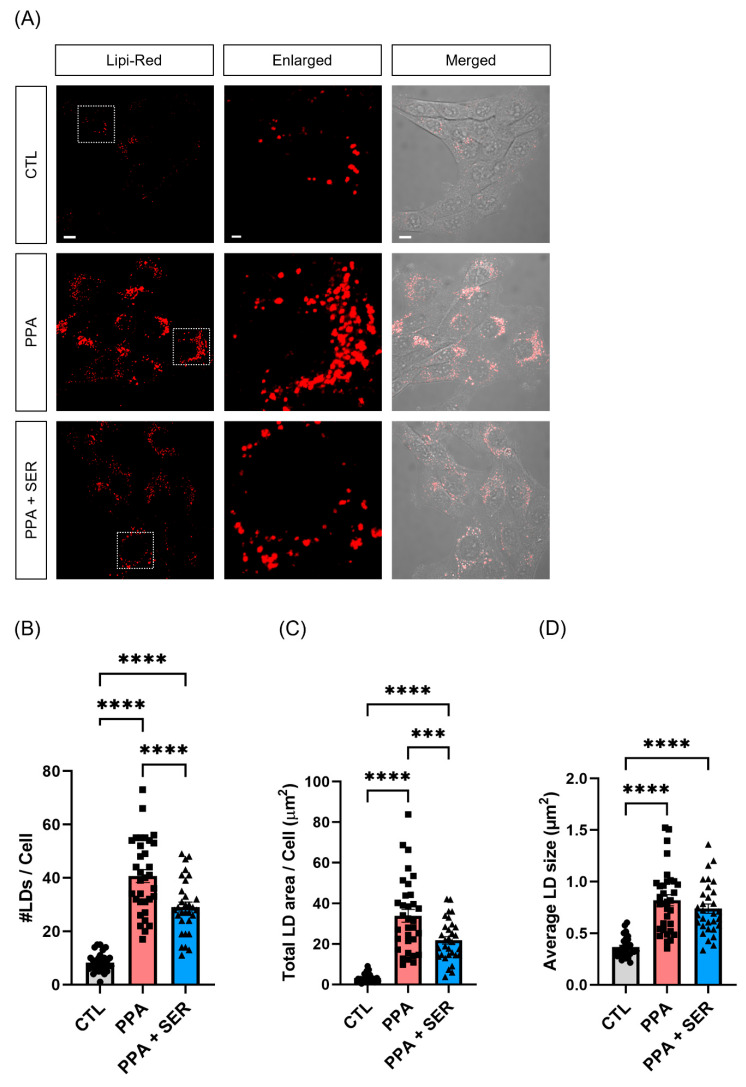
PPA induced lipid droplet (LD) accumulation in HT22 cells. Cells were treated with 5 mM PPA only or 5 mM PPA and 10 mM L-serine (SER) for 48 h. (**A**) Confocal imaging of HT22 incubated with red fluorescence to label lipid droplets. Scale bar, 10 µm (left, right); 2 µm (middle, the magnified images). (**B**–**D**) Quantification of Lipi-red number of puncta (**B**), total area (**C**), and average size (**D**) (*n* = 30). *** *p* < 0.001, **** *p* < 0.0001 by One-way ANOVA, Tukey’s multiple-comparison test.

Some studies have provided evidence that lipid droplet formation is significantly stimulated by SCFAs [23]. To investigate whether PPA affected lipid droplets, we treated HT22 cells with PPA only or PPA together with L-serine. After treatment for 48 h, we stained the cells with lipi-red probe and incubated them for 30 min. We then measured the red fluorescent puncta using confocal microscopy (Figure 3A). The number and total area of lipid droplets were significantly increased in HT22 cells treated with PPA (Figure 3B,C), and the average size of lipid droplets was significantly increased compared with the control group (Figure 3D). In contrast, co-treatment with PPA and L-serine significantly decreased the number and total area of lipid droplets compared with cells treated only with PPA. In this study, we found that PPA induced lipid droplet accumulation in HT22 cells.

### 2.4. Three-Dimensional Volume Reconstruction in Serial TEM Showed Effects of L-Serine in Decreasing Lipid Droplets in PPA-Treated HT22 Cells

**Figure 4:**
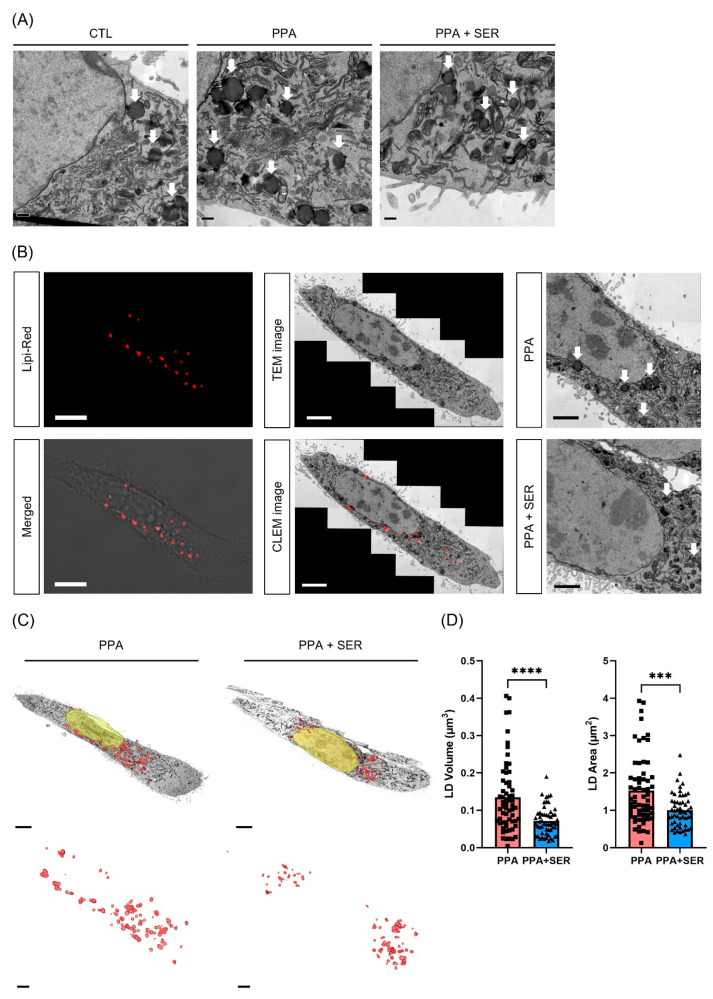
Three-dimensional volume reconstruction of lipid droplets (LD) shows a different size in PPA-treated HT22 cells. Cells were treated with 5 mM PPA only or 5 mM PPA with 10 mM L-serine (SER) for 48 h. (**A**) Representative electron microscopic images of HT22 cells. TEM images showed the increased size of lipid droplets in PPA-treated HT22 cells. White arrows represent lipid droplets (LD). Scale bar, 1 μm. (**B**) Representative CLEM images of lipid droplets (LD) in PPA-treated HT22 cells. The location of the red signal under confocal microscopy was specifically in the abnormal lipid droplets (LD) under electron microscopy. Scale bar, 10 μm (confocal image); 5 μm (TEM image). (**C**) Representative 3D volume reconstruction of lipid droplets (LD) in HT22 cells. Scale bar, 4 μm (top); 2 μm (bottom, the magnified images). (**D**) Quantification of 3D volume and area of reconstructing lipid droplets (LD) in HT22 cells. *** *p* < 0.001, **** *p* < 0.0001 by unpaired two-samples *t*-test.

TEM showed that the lipid droplets in HT22 cells were larger in the presence of PPA (Figure 4A). We also observed the location of lipid droplet accumulation using CLEM (Figure 4B). CLEM is a powerful technique that provides both the valuable functional information given by fluorescence microscopy and the high-resolution structural information given by TEM [24]. The use of this technique allowed us to observe more clearly the location of lipid droplets using lipi-red fluorescence and the detail structures in cells under electron microscopy. We acquired serial sections and images for three-dimensional (3D) volume reconstruction, and the 3D structure was visualized with Amira software (Figure 4C). Structural analysis through 3D reconstruction revealed a significantly decreased volume and area of lipid droplets in cells co-treated with PPA and L-serine than in cells treated only with PPA (Figure 4D). These data suggested that L-serine decreased the formation and accumulation of lipid droplets induced by PPA.

### 2.5. L-Serine Decreased the Colocalization of Lipi-Red and Lysotracker in PPA-Treated Hippocampal Neurons

**Figure 5:**
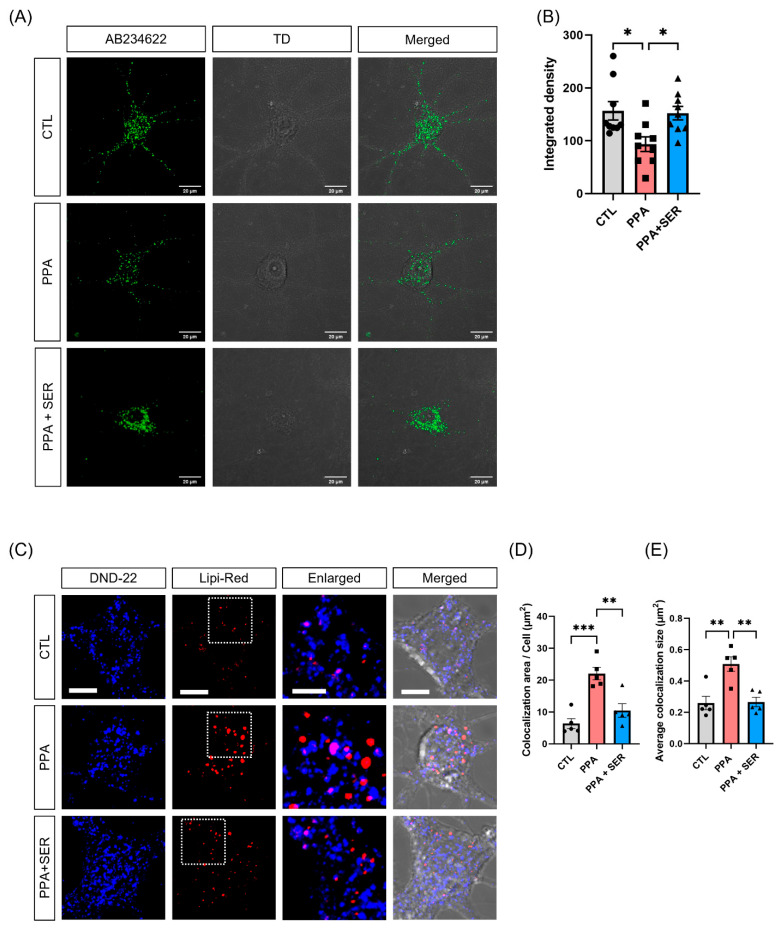
L-serine (SER) reduced the colocalization of lysosomes and lipid droplets against PPA. (**A**) Representative fluorescence images of the release of the self-quenched substrate in hippocampal neurons (*n* = 8). Confocal images showed lysosomal activity with green fluorescence. Scale bar, 20 μm. (**B**) Quantification of the integrated density in hippocampal neurons. PPA significantly reduced lysosomal activity compared to control. L-serine (SER) significantly increased lysosomal activity compared to PPA. * *p* < 0.05 by one-way ANOVA, Tukey’s multiple-comparison test. TD: transmitted light detector image. AB234622: Lysosomal intracellular activity assay kit. (**C**) Representative fluorescence images of colocalization of lysosome and lipid droplets (LD) in hippocampal neurons. Blue fluorescent signals represent lysosomes, and red fluorescent signals represent lipid droplets (LD). Scale bar, 10 µm; 5 µm (the magnified images). (**D**,**E**) Quantification of total area (**D**) and average size (**E**) of colocalization of lysosomes and lipid droplets (LD) in hippocampal neurons. ** *p* < 0.01, *** *p* < 0.001 by one-way ANOVA, Tukey’s multiple-comparison test.

To confirm whether PPA induces lysosomal dysfunction in hippocampal neurons, we performed an intracellular lysosomal activity assay in hippocampal neurons treated with PPA or co-treated with PPA and L-serine. We measured lysosomal activity using confocal microscopy. Green puncta represent the lysosomal activity (Figure 5A, Supplementary Figure S1A). As shown in Figure 5B, hippocampal neurons treated only with PPA showed significantly decreased lysosomal activity compared to the control group. However, neurons co-treated with PPA and L-serine had significantly increased lysosomal activity compared with PPA-only treated hippocampal neurons. These results showed that PPA induced lysosomal dysfunction, and L-serine increased lysosomal activity in hippocampal neurons as in HT22 cells.

To investigate whether dysfunctional lysosomes do not degrade abnormally accumulated lipid droplets, we measured the colocalization of lipi-red and DND-22 lysotracker signal in hippocampal neurons (Figure 5C, Supplementary Figure S1B). The colocalization of total area in each group was 6.3676 ± 3.4905474 µm^2^ (control group), 22.0612 ± 4.4119301 µm^2^ (PPA group) and 10.4798 ± 4.7557928 µm^2^ (PPA + SER group) in hippocampal neurons. The colocalization of average puncta size in each group was 0.2592 ± 0.097189 µm^2^ (control group), 0.5084 ± 0.103289883 µm^2^ (PPA group) and 0.2658 ± 0.0679169 µm^2^ (PPA + SER group) in hippocampal neurons. Colocalization of the total area and average puncta size were significantly increased compared to control in PPA-treated hippocampal neurons. The colocalization of the total area and the average size were significantly decreased by co-treatment with PPA and L-serine (Figure 5D,E). These data suggested that L-serine decreased the accumulation of lipid droplets induced by PPA through the increase in lysosomal activity. 

### 2.6. Attenuation of Lysosomal Dysfunction by L-Serine in PPA-Treated Hippocampal Neurons

**Figure 6:**
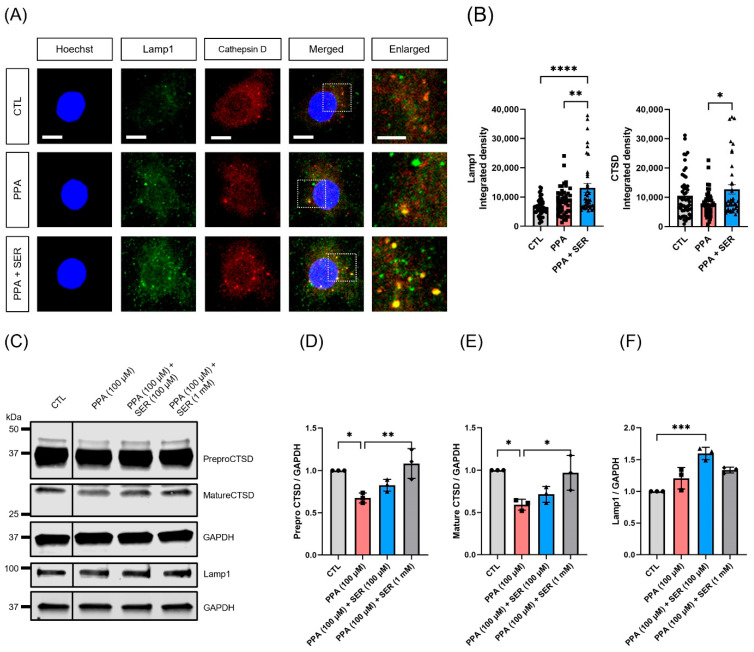
L-serine (SER) increased lysosomal activity in hippocampal neurons. (**A**) Representative lamp1 and CTSD immunocytochemistry images of rat hippocampal neurons. Cultured neurons were treated at DIV 18 with 100 μM PPA or 100 μM PPA and 100 μM L-serine for 48 h, and immunolabelled for lamp1 or CTSD. Scale bar, 10 μm; 5 μm (right, the magnified images). (**B**) Quantification of the integrated density of lamp1 or CTSD in hippocampal neurons (*n* = 40). (**C**) Representatives immunoblot of lamp1 and CTSD. (**D**,**E**) Analysis of CTSD protein expression by quantification of CTSD levels normalized to GAPDH. (**F**) Analysis of Lamp1 protein expression by quantification of lamp1 levels normalized to GAPDH. Data obtained from independent experiments (*n* = 3 replicates) are presented as the mean ± SEM. * *p* < 0.05, ** *p* < 0.01, *** *p* < 0.001, **** *p* < 0.0001 by one-way ANOVA, Tukey’s multiple-comparison test.

To further examine whether L-serine affects lysosomal activity in PPA-treated hippocampal neurons, we performed immunocytochemistry with the lysosome marker lamp1 and the active lysosome marker cathepsin D (CTSD) (Figure 6A, Supplementary Figure S2). Co-treatment of hippocampal neurons with PPA and L-serine significantly increased the fluorescence intensity of lamp1 and CTSD compared to treatment only with PPA (Figure 6B). We also examined the expression levels of lamp1 and CTSD in each group of hippocampal neurons with treatments (Figure 6C). The quantitation of CTSD band intensity normalized to housekeeping GAPDH signals indicated a significant increase in the level of CTSD in hippocampal neurons co-treated with PPA and L-serine. Compared to control, CTSD levels were downregulated in hippocampal neurons treated only with PPA (Figure 6D,E). The expression level of prepro CTSD (fold change) in each group was 0.675844667 ± 0.059133796 (PPA 100 µM), 0.826306333 ± 0.06993275 (PPA 100 µM + SER 100 µM) and 1.082893333 ± 0.176601 (PPA 100 µM + SER 1 mM). The expression level of mature CTSD (fold change) in each group was 0.591488 ± 0.064692 (PPA 100 µM), 0.714738 ± 0.093947 (PPA 100 µM + SER 100 µM) and 0.969378 ± 0.205194 (PPA 100 µM + SER 1 mM). Co-treatment of hippocampal neurons with PPA and L-serine showed an increased level of lamp1 compared to that in cells treated only with PPA (Figure 6F). The expression level of lamp1 (fold change) in each group was 1.206469 ± 0.169617 (PPA 100 µM), 1.597523 ± 0.096476 (PPA 100 µM + SER 100 µM) and 1.335504 ± 0.045853 (PPA 100 µM + SER 1 mM). These results provided further evidence that L-serine increased lysosomal activity in PPA-treated hippocampal neurons through regulation of CTSD levels [25].

### 2.7. CLEM and Serial TEM Showed Increase in Lysosomal Lipid Membrane Accumulation by PPA in Hippocampal Neurons

**Figure 7:**
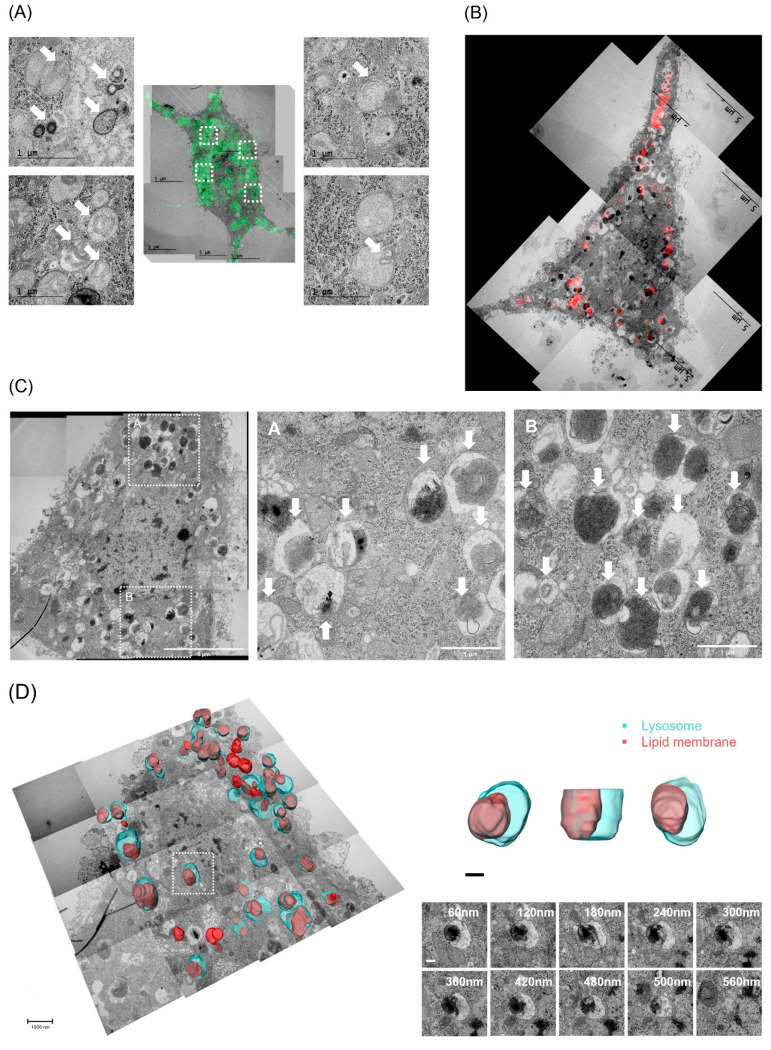
PPA induced lipid membrane accumulation inside the lysosomes in hippocampal neurons. Representative CLEM images of the lamp1 (**A**) or lysotracker (**B**) in PPA-treated hippocampal neurons. The white arrow indicates the lipid membrane accumulation inside the lysosomes. Scale bar, 1 µm (left); 5 µm (right). (**C**) Representative TEM images of lipid membrane accumulation inside the lysosomes in the presence of PPA in hippocampal neurons. Scale bar, 5 µm (left); 1 µm (the magnified images). The white arrow indicates the lipid membrane accumulation inside the lysosomes. (**D**) Representative 3D volume reconstruction images in PPA-treated hippocampal neurons. Aqua color, lysosome; crimson color, lipid membrane accumulation. Scale bar, 1 µm (left); 200 nm (right); 100 nm (bottom).

Finally, to confirm whether lipid membranes are accumulated inside the abnormal lysosome in hippocampal neurons by PPA, we examined the lysosomal location and morphology in hippocampal neurons using TEM and immunofluorescence staining with lamp1 and lysotracker. The number of puncta identified in cells transfected with lamp1-GFP was increased in PPA-treated hippocampal neurons (Figure 7A). CLEM analysis revealed abnormally increased sizes of lysosomes in hippocampal neurons treated with PPA (Figure 7B). TEM images also showed an increase in abnormally large vacuoles and accumulation of lipid membranes inside the lysosomes (Figure 7C). Accumulated lipid membranes were observed more clearly inside the lysosomes using 3D volume reconstruction analysis. Lysosomal lipid membrane accumulation was further confirmed by the serial section images (Figure 7D).

## 3. Discussion

In this study, we observed the accumulation of lipid droplets and lysosomal lipid membranes in PPA-treated hippocampal neuronal cells (Figure 3 and Figure 7). Recent studies have demonstrated that changes in lipid metabolism and the rearrangement of cellular lipid composition are important in the adaptation of yeast cells to acid stress. These lipid rearrangements include changes in the abundance of sphingolipids, changes in the quantity of sterol, and adaptations in fatty acid saturation and chain length [26,27,28,29]. PPA and butyrate significantly stimulated the triacylglycerol content and extent of lipid droplet formation [30,31]. SCFAs affect the mRNA expression level of lipogenic genes. The expression of fatty acid binding protein 3 (FABP3), stearoyl-CoA desaturase-1 (SCD1), peroxisome proliferator-activated receptor (PPARG), sterol regulatory elements-binding transcription factor 1 (SREBP1), diacylglycerol acyltransferase 1 (DGAT1), l-acylglycerol-3-phosphate O-acyltransferase 6 (AGPAT6), and adipose differentiation-related protein (ADFP) was upregulated by PPA and butyrate treatment. Besides the suggestion that PPA can affect the expression of SCD1 and FABP3 through PPARG, the upregulation of SREBP1 by PPA suggests that PPA regulates the desaturation and transport of fatty acids through the combined action of PPARG and SREBP1 [23]. Our results also showed the abnormal structure of lysosomes and a decrease in lysosomal activity in PPA-treated hippocampal neuronal cells (Figure 4). Lysosomes are dynamic organelles that respond to cellular conditions, and lysosomal activity correlates with physical changes in lysosomal structure, including their size, number, and position [32]. The size of a lysosome regulates its dynamics, and its relationship with several human diseases has been reported. Luminal pH is closely related to the functions of organelles. Cytoplasmic acidification regulates the localization and shape of lysosomes closer to plasma membranes [33]. Deacidification changes the position of endolysosomes in the cytoplasm [34]. It affects the intraluminal and cytosolic levels of divalent cations, the expression levels and activity of acid hydrolases, endolysosome volumes and sizes, and various protein aggregations [35,36]. Lysosomal pH is regulated by vacuolar-type ATPase (V-ATPase) through the transport of protons into the lysosomal lumen. V-ATPase dysfunction influences the lysosomal pH, disrupting substrate degradation and leading to neurodegenerative diseases [37]. One of the effects of lysosomal dysfunction induced by PPA appears to be changed in intraluminal pH and subsequently the acidification of the lysosomes. PPA is the anion of a weak acid, which is known to acidify the interior of cells. The undissociated form of PPA enters the cytoplasm and dissociates, thus decreasing the intracellular pH. Since PPA affects intracellular acidification, V-ATPase acts as a key player in counteracting changes in intracellular pH and membrane proton gradients. V-ATPase can change the structure in vivo in response to various stimuli, including increased glucose concentrations, amino acid starvation, and growth factors. For example, the binding of concanamycin, a V-ATPase inhibitor, to the pump may hinder the interactions necessary for amino acid sensing [38]. Starvation of some amino acids, including serine, glutamate, and aspartate, leads to a decrease in V-ATPase activity [39]. Many V-ATPase inhibitors, including archazolid, the plant toxin pea albumin 1 subunit b (PA1b), and concanamycin have binding sites that overlap with bafilomycin, indicating a common mechanism of inhibition [40]. The macrolide bafilomycin A1 is synthesized starting from D-valine and D-mannitol as chiral progenitors of propionate units [41]. Due to a lack of tissue specificity, most available V-ATPase inhibitors cause cellular toxicity [42]. For these reasons, PPA may have a functional effect similar to that of the lysosomal inhibitor Bafilomycin A1, which may degrade lysosomal activity. The lysosomal proteases carry out the degradation function of the lysosomes within their lumen. These enzymes are active under highly acidic pH in the luminal compartment. The lysosomal protease CTSD was reported to protect against alpha-synuclein aggregation and toxicity in mouse models [43,44,45]. A decreased level of CTSD was found in several models of neurodegeneration and aged rats [46]. L-serine selectively activates lysosomal cathepsins B and L, and it has been suggested that cysteine protease mediates the processing of CTSD, and cysteine cathepsins B and L are involved in the final processing step. This may be a helpful pharmacological mediator for the regulation of autophagy [47].

Our results showed that L-serine co-treatment increases the degradative function of lysosomes against PPA-treated hippocampal neurons (Figure 4). The level of CTSD was significantly increased by L-serine compared to that in PPA-treated cells (Figure 6). Endogenous CTSD inhibitors are unknown, and pH seems to be the major factor regulating mature CTSD activity. CTSD is mainly activated at low pH, such as in lysosomes, because the two active sites, aspartic residues, are prone to deprotonation [48]. Therefore, PPA may affect the activity of CTSD, as well as the level of CTSD, by changing the intracellular pH. In this study, we identified an accumulation of lipid membranes in PPA-treated hippocampal neurons. Inhibition of CTSD was affected in cholesterol metabolism and degradation in murine steatohepatitis. Inhibition of CTSD activity dramatically improved lipid metabolism in hepatic inflammation [49]. CTSD activity might affect cellular cholesterol transport or efflux. CTSD binding of ceramide affects its autocatalytic activity. In cells with acid sphingomyelinase deficiency, low endogenous ceramide is associated with decreased CTSD activity. CTSD regulates the intracellular transport of phospholipids, cholesterol, and ATP-binding cassette protein A1 (ABCA1)-mediated lipid efflux [50]. The role of ABC proteins in many species is known to involve maintaining the low pH of organelles. V-ATPase is recruited to the cell surface by ABCA1 without affecting total V-ATPase levels, and inhibition of V-ATPase significantly impairs ABCA1-mediated cholesterol efflux [51].

In summary, our results demonstrate that PPA induced a decrease in lysosomal activity and accumulation of lipid droplets. These effects may be the results of changes in intracellular pH, which affect lysosomal activity, and PPA may, therefore, have potential as a V-ATPase inhibitor. PPA may also increase the mRNA expression level of lipogenic genes. L-serine can be a neuroprotective agent that restores the lysosomal damage caused by PPA. L-serine may increase the lipid efflux of ABCA1 by increasing the assembly of V-ATPase. In addition, the degradation of lipid droplets may be increased because of the increase in lysosomal activity. Therefore, our results demonstrate that L-serine may be a major player in lipid metabolism, regulating lysosomal activity in hippocampal neuronal cells.

## 4. Materials and Methods

### 4.1. Cell Culture

The hippocampal neuronal cell line HT22 was maintained in Dulbecco’s modified Eagle’s medium (#11995-065, Gibco, Waltham, MA, USA) supplemented with 10% (*v*/*v*) FBS, 20 mM HEPES (pH 7.2), and 1% penicillin-streptomycin (#15140122, Gibco, Waltham, MA, USA) in a CO_2_ incubator at 37 °C. Primary hippocampal neurons were cultured from embryonic day 18 Sprague–Dawley fetal rats, as previously described [52]. Briefly, the hippocampal tissue was dissected in Hanks’ Balanced Salt solution (#14185052, Gibco, Waltham, MA, USA) and dissociated with 0.125% trypsin (#15090046, Gibco, Waltham, MA, USA) for 15 min at 37 °C. Cells were seeded on a pre-coated coverslip or plate with 100 μg/mL poly-D-lysine (#P6407, Sigma-Aldrich, St. Louis, MO, USA) and 10 μg/mL laminin (#354232, Corning, Lowell, MA, USA). The hippocampal neurons were incubated in a Neurobasal medium (#21103-049, Gibco, Waltham, MA, USA), 0.5 mM glutamine, and 25 μM glutamate and supplemented with SM1 components (#05711, Stemcell, Vancouver, BC, Canada) for 18–21 days in vitro.

### 4.2. Pharmacological Treatment

Propionic acid (#402907, Sigma-Aldrich, St. Louis, MO, USA) was purchased from Sigma-Aldrich, and L-serine was provided by Astrogen Ltd., Daegu, Korea. PPA and L-serine were dissolved in cell culture grade water (LS 016-01, Welgene, Gyeongsan, Korea) for treatment at each concentration.

### 4.3. Cell Viability Assays

For the cell viability assays, HT22 cells (2 × 10^3^/well) were added into 96-well plates with three wells for each condition, in DMEM medium with 10% FBS and 1% P/S. Cells were cultured overnight, and the following day they were incubated with PPA or L-serine at concentrations of 0, 1, 2, 5, 10, and 20 mM for 48 h followed by CCK-8 assay (Chromo-CK, #CH-3000, Monobio, Seoul, Korea). In order to investigate the protective effects of L-serine, cells incubated with the six concentrations of PPA were then treated with 5 mM or 10 mM L-serine. The OD values at a wavelength of 450 nm were measured using a spectrophotometer (VersaMax, Sunnyvale, CA, USA).

### 4.4. Trypan Blue Staining

Cultured cells were fixed with 4% paraformaldehyde in PBS for 20 min at room temperature, and then washed twice with PBS. The fixed cells were then stained with 0.4% Trypan Blue Stain (#15250061, Gibco, Waltham, MA, USA) for 30 min at room temperature. The cells were then washed to completely remove the remaining trypan blue residue. Optical images were acquired using a 10 × inverted microscope (Eclipse TS100, Nikon, Tokyo, Japan) with a TUCSEM ISH300 camera.

### 4.5. Lysosomal Activity Assays

The lysosomal activity was confirmed using Lysosomal Intracellular Activity Kits (ab234622, Abcam, Cambridge, UK) following the manufacturer’s instructions. Lysosome-specific self-quenched substrate has low background fluorescence and a high signal to background ratio. The substrate, acting as endocytic cargo, can be taken up by cells and degraded within an endo-lysosomal vesicle. The fluorescence signal generated by degradation is proportional to the intracellular lysosomal activity. Briefly, neuronal cells were incubated in the presence of only 5 mM PPA, 10 mM L-serine with 5 mM PPA, or without any substrate as controls. The cells were cultured in appropriate density and treated with PPA or co-treated with PPA and L-serine for 48 h. The next day, the medium was removed and replaced with fresh medium containing the same concentration of PPA or PPA + L-serene, and the cells were incubated for 1 h in a CO_2_ incubator at 37 °C. Then the medium was removed and replaced with fresh medium supplemented with 0.5% FBS. Self-quenched substrate was added, and the cells were incubated for 1 h. After incubation, the cells were washed twice in 1 mL ice-cold 1 × assay buffer in the presence of PPA or PPA + L-serine at the same concentration. Finally, the cells were observed under fluorescence confocal microscope with a 488 nm excitation filter. The confocal microscopic images were acquired using an A1 Rsi/Ti-E confocal microscope (Nikon, Tokyo, Japan) with a 60 × oil-immersion lens. Z-stack image sequences were acquired at 0.4 μm intervals for a total of 11 images and converted with maximal intensity projection using NIS-element AR software (Nikon, Tokyo, Japan). Integrated density was analyzed using the ImageJ software.

### 4.6. Immunocytochemistry

Primary hippocampal neurons were fixed with 4% paraformaldehyde in PBS with 4% sucrose for 15 min at room temperature for immunofluorescent labeling of endogenous LAMP1 (H4A3, Santa Cruz, Heidelberg, Germany) and cathepsin D (#69854, Cell Signaling Technology, Danvers, MA, USA). Cultured neurons were incubated with lamp1 or cathepsin D antibody in a GDB solution (450 mM NaCl, 0.1% gelatin, 0.3% Triton X-100, and 30 mM phosphate buffer, pH 7.4) overnight at 4 °C. After being washed with PBS, coverslips were incubated with AlexaFluor488 (#4412, Cell Signaling Technology, Danvers, MA, USA) or AlexaFluor594 (#8890, Cell Signaling Technology, Danvers, MA, USA)-conjugated secondary antibodies for 1 h at room temperature. After washing with PBS and distilled water, the cells were stained with 5 μg/mL Hoechst 33342 (#H1399, Thermo Fisher Scientific, Waltham, MA, USA) to label the nuclei. Finally, coverslips were mounted with VECTASHIELD mounting solution (H-1000, Vector Laboratories Burlingame, CA, USA).

### 4.7. Confocal Microscopy Analysis

To visualize the accumulation of lipid droplets, cells were stained with 1 μM Lipi-Red probe (LD03-10, Dojindo, Kumamoto, Japan) for 30 min. To confirm the colocalization of lipids and lysosomes, the cells were stained with 100 nM Lysotracker Blue DND-22 (L7525, Thermo Fisher Scientific, Waltham, MA, USA) or 100 nM Lysotracker DND-99 (L7528, Thermo Fisher Scientific, Waltham, MA, USA) for 15 min and 1 μM Lipi-Red probe (LD03-10, Dojindo, Kumamoto, Japan) for 30 min. To confirm the location of lysosomes, the hippocampal neurons were transfected with 2 μg of lamp1-mGFP (#34831, Addgene, Watertown, MA, USA) with 2 μL of lipofectamine 2000 reagent (Thermo Fisher Scientific, Waltham, MA, USA). The cells were then stained with anti-GFP antibody (A11122, Invitrogen, Carlsbad, CA, USA). The confocal microscopic images were acquired using an A1 Rsi/Ti-E confocal microscope (Nikon, Tokyo, Japan) with a 60 × oil-immersion lens. Z-stack image sequences were acquired at 0.4 μm intervals and converted with maximal intensity projection using NIS-element AR software (Nikon, Tokyo, Japan).

### 4.8. Western Blotting Analysis

Cultured cells were lysed with RIPA buffer (R0278, Sigma-Aldrich, St. Louis, MO, USA) containing phosphatase inhibitor (P3200, GenDEPOT, Katy, TX, USA) and protease inhibitor (P3100, GenDEPOT, Katy, TX, USA). After incubation on ice for 30 min, all lysates were centrifuged at 15,000× *g* at 4 °C for 30 min. Total protein concentrations were evaluated using a BCA Protein Assay Kits (#23225, Thermo Fisher Scientific, Waltham, MA, USA). Equal amounts of protein samples were incubated with 4 × Laemmli sample buffer (#1610747, Bio-Rad, Hercules, CA, USA) at 95 °C for 5 min. The samples were separated on 4–15% polyacrylamide gels (#4561084, Bio-Rad, Hercules, CA, USA) and transferred to a nitrocellulose membrane (Whatman, Maidstone, UK). The membranes were blocked using 3% BSA (#9048-4-8, GENEray Biotechnology, Shanghai, China) and 0.1% Tween 20 (H5152, Promega, Madison, WI, USA) in TBST buffer (#CBTB-9110, CHEMBIO, Seoul, Korea) for 1 h and washed with TBST. Blots were incubated with lamp1 (H4A3, Santa Cruz, Heidelberg, Germany) or cathepsin D (#69854, Cell Signaling Technology, Danvers, MA, USA) at 4 °C overnight, followed by infrared dye-conjugated secondary antibodies (Li-Cor Bioscience, Lincoln, NE, USA) for 1 h. Then, blots were detected using the Odyssey CLx Infrared Imaging System (Li-Cor Bioscience, Lincoln, NE, USA). Blot images were analyzed using the ImageJ software (National Institutes of Health).

### 4.9. Transmission Electron Microscopy (TEM)

The cells were grown in 35 mm glass grid-bottomed culture dishes (P35G-1.5-14-C-GRID, MatTek, Ashland, MA, USA) to 50–60% confluency. After acquiring confocal images, the cells were prefixed with 2% paraformaldehyde and 2.5% glutaraldehyde in 0.1 M sodium cacodylate buffer (pH 7.4). After being washed three times in 0.15 M cacodylate buffer, cells were postfixed in 2% osmium tetroxide (#1610747, EMS, Hatfield, PA, USA) with 1.5% ferrocyanide in 0.1 M sodium cacodylate buffer on ice for 1 h. After washing three times in ddH_2_O for 5 min, the cells were incubated in 1% thiocarbohydrazide (#T1136, TCI, Tokyo, Japan) for 20 min at room temperature. After being washed three times in ddH_2_O at room temperature for 5 min, the cells were placed in 2% OsO_4_ in ddH_2_O for 30 min at room temperature. After being washed in ddH_2_O at room temperature for 30 min, they were en bloc stained with 1% uranyl acetate overnight at 4 °C. The cells were then washed three times in ddH_2_O for 5 min. For embedding, samples were dehydrated through a graded ethanol series (20, 30, 50, 70, 80, 90, and 100% for 10 min each, all cooled to 4 °C) followed by epoxy resin infiltration by immersion into 3:1, 1:1, and 1:3 mixtures of ethanol and Epon 812 resin (EMS, Hatfield, PA, USA) at room temperature for 2 h. The infiltrated samples were then incubated in pure resin overnight and placed in an inverted capsule on a confocal dish in a pre-warmed oven (70 °C) for 48 h. After trimming the epoxy resin embedded cells, ultrathin serial sections 60 or 100 nm thick were cut using an ultramicrotome (EM UC7, Leica, Wetzlar, Germany) and mounted on 0.5% formvar coated on hole grids. To enhance the electron density, the sections were stained with UranylLess (#22409, EMS, Hatfield, PA, USA) and 3% lead citrate (#22410, EMS, Hatfield, PA, USA). TEM images were acquired using a Tecnai G2 20 (Thermo Fisher Scientific, Waltham, MA, USA) at 120 kV with a US1000XP CCD detector (Gatan, Pleasanton, CA, USA).

### 4.10. Three-Dimensional Reconstruction of Serial TEM Images

Serial TEM images were aligned using the TrakEM2 plugin in Fiji/ImageJ. The montaged images were checked visually, and the stack slice images were aligned manually using a landmark point. Subsequently, the corrected image stacks were imported into Amira software (Thermo Fisher Scientific, Waltham, MA, USA). The nucleus and lipid droplets were selected by tracking their membrane boundaries. After identifying the organelle structures in the serial images, 3D surface-rendering reconstruction was performed using Amira.

### 4.11. Statistical Analysis

Statistical analyses were performed using GraphPad Prism 8.0 (GraphPad Software, San Diego, CA, USA, www.graphpad.com). Data are shown as mean ± standard deviation or SEM. Statistical significance was calculated using unpaired two-sample *t*-tests and Turkey’s multiple-comparisons tests. Statistically significant differences were determined as * *p* < 0.05.

## 5. Conclusions

In this paper, lysosome dysfunction caused by PPA, which was known from previously published results, was confirmed in primary hippocampal neurons and HT22 hippocampal neurons. Specifically, the morphology of the lipid membrane, which was not degraded in lysosomes, was observed, which could be elucidated using the advantages of electron microscopy and CLEM. As a result of examining the difference in lysosome protein expression level, we can suggest that the morphological changes occur because of CTSD levels. This means that PPA inhibits the expression level of CTSD to lower the lysosome activity. In both systems using primary hippocampal neurons and HT22 cells, L-serine increased the lysosome activity by increasing the expression level of CTSD in PPA treated cells. Therefore, these data suggest that L-serine has protective potentials against PPA-induced functional and structural changes of lysosomes by restoring CTSD levels in hippocampal neurons.

## Data Availability

The data that support the findings of this study are available from the corresponding author upon reasonable request.

## Supplementary Materials

The following supporting information can be downloaded at: https://www.mdpi.com/article/10.3390/ijms231810613/s1.

## Abbreviations

PPA	propionic acid
SER	L-serine
CTSD	cathepsin D
LD	lipid droplet
SCFAs	short-chain fatty acids
TEM	transmission electron microscopy
CLEM	correlative light and electron microscopy
V-ATPase	vacuolar-type ATPase
AB234622	lysosomal intracellular activity assay kit
