# Supplementary material for: Restoration of Cathepsin D Level via L-Serine Attenuates PPA-Induced Lysosomal Dysfunction in Neuronal Cells
Source: Int J Mol Sci. 2022 Sep 13;23(18):10613. doi: 10.3390/ijms231810613 (PMC9504002; doi:10.3390/ijms231810613)
Supplement: Supplementary file 1 [file ijms-23-10613-s001.zip › ijms-1864568-supplementary.pdf]

Supplementary Information

Restoration of Cathepsin D Level via L-Serine Attenuates PPA-Induced Lysosomal Dysfunction in Neuronal Cells

Hyunbum Jeon 1,2, Yeo Jin Kim 1 , Su-Kyeong Hwang 3,4, Jinsoo Seo 2 and Ji Young Mun 1,*

1 Neural Circuit Research Group, Korea Brain Research Institute, Daegu 41062, Korea
2 Department of Brain Sciences, Daegu Gyeongbuk Institute of Science and Technology (DGIST), Daegu 42988, Korea
3 Department of Pediatrics, School of Medicine, Kyungpook National University, Daegu 41944, Korea
4 Astrogen Inc., 440, Hyeoksin-daero, Dong-gu, Daegu 41072, Korea
* Correspondence: Correspondence: jymun@kbri.re.kr

Supplementary Figures

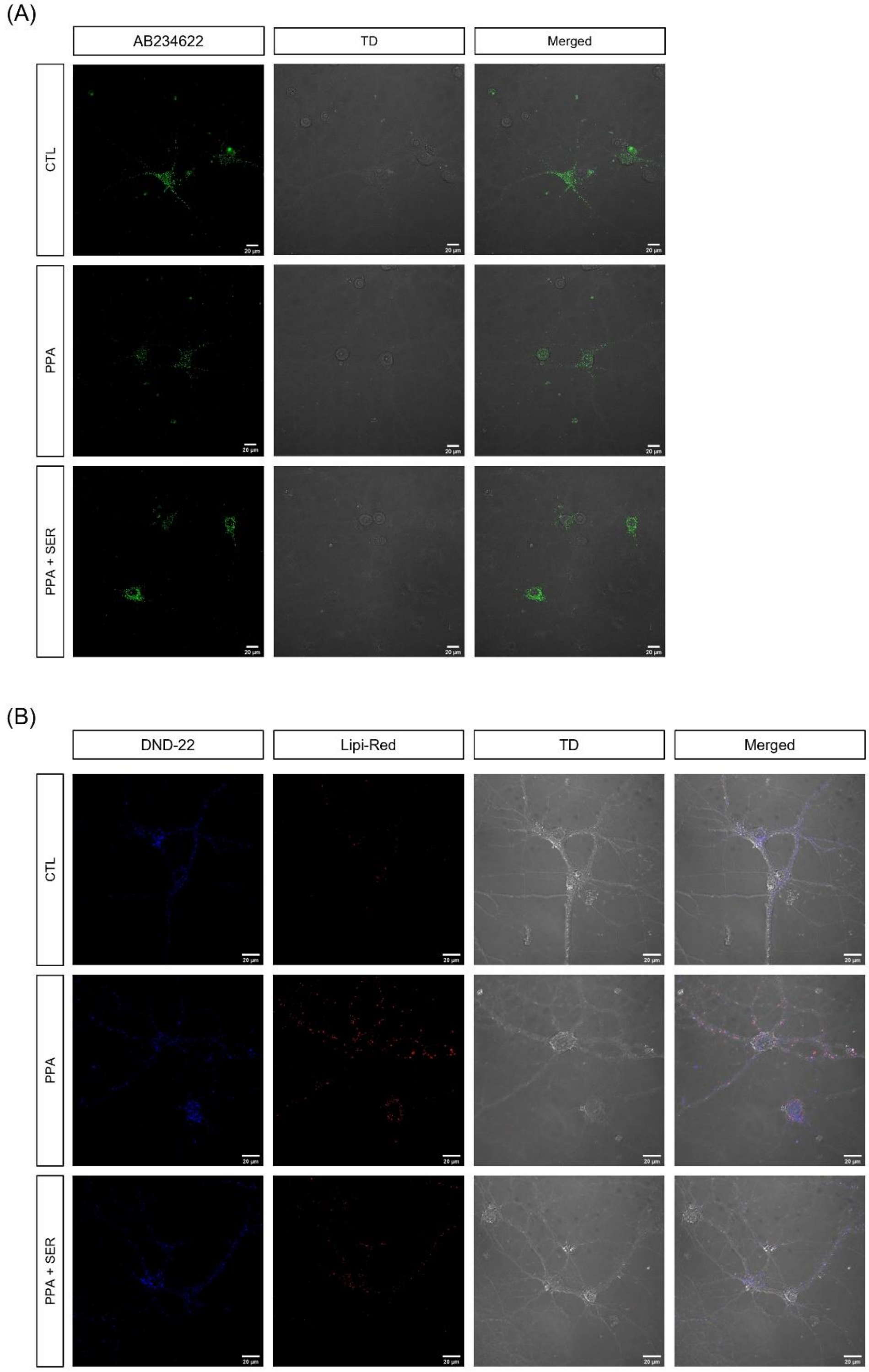

Supplementary Figure S1. L-serine (SER) reduced the colocalization of lysosomes and lipid droplets against PPA. (A) Representative fluorescence images of the release of the self-quenched substrate in hippocampal neurons. Confocal images showed lysosomal activity with green fluorescence. Scale bar, 20 μm. TD : transmitted light detector image. AB234622 : Lysosomal intracellular activity assay kit. (B) Representative fluorescence images of colocalization of lysosome and lipid droplets (LD) in hippocampal neurons. Blue fluorescent signals represent

lysosomes and red fluorescent signals represent lipid droplets (LD). Scale bar, 20 µm.

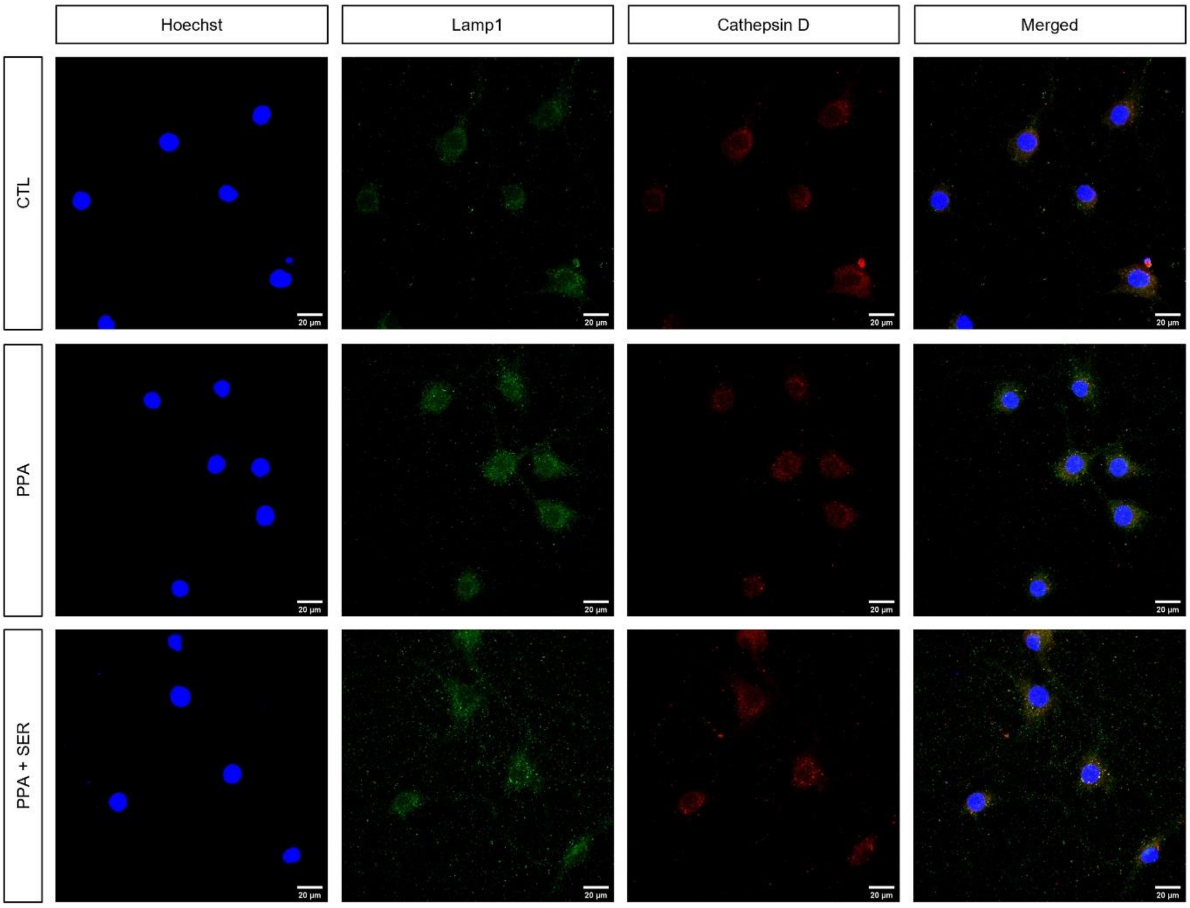

Supplementary Figure S2. L-serine (SER) increased lysosomal activity in hippocampal neurons. Representative lamp1 and CTSD immunocytochemistry images of rat hippocampal neurons. Cultured neurons were treated at DIV 18 with 100 µM PPA or 100 µM PPA and 100 µM L-serine for 48 h, and immunolabelled for lamp1 or CTSD. Scale bar, 20 µm.

Supplementary Table S1

Figure 2. **(B)**

Group	Summary	*p* value	
CTL vs. PPA	****	<0.0001	0.000079
CTL vs. PPA + SER	ns	ns	0.94269
PPA vs. PPA + SER	***	<0.001	0.000223

(D)

Group	Summary	*p* value	
CTL vs. PPA	*	<0.05	0.0163
CTL vs. PPA + SER	ns	ns	0.6164
PPA vs. PPA + SER	ns	ns	0.0883

Figure 3. **(B)**

Group	Summary	*p* value	
CTL vs. PPA	****	<0.0001	5E-10
CTL vs. PPA + SER	****	<0.0001	5E-10
PPA vs. PPA + SER	****	<0.0001	7.01145E-05

(C)

Group	Summary	*p* value	
CTL vs. PPA	****	<0.0001	5E-10
CTL vs. PPA + SER	****	<0.0001	1.863E-07
PPA vs. PPA + SER	***	<0.001	0.000511297

(D)

Group	Summary	*p* value	
CTL vs. PPA	****	<0.0001	6E-10
CTL vs. PPA + SER	****	<0.0001	6.88E-08
PPA vs. PPA + SER	ns	ns	0.378272859

Figure 4. **(D) LD volume**

Group	Summary	*p* value	
PPA vs. PPA + SER	****	<0.0001	9.5753E-06

(D) LD area

Group	Summary	*p* value	
PPA vs. PPA + SER	***	<0.001	0.000144046

Figure 5. **(B)**

Group	Summary	*p* value	
CTL vs. PPA	*	<0.05	0.0155
CTL vs. PPA + SER	ns	ns	0.9758
PPA vs. PPA + SER	*	<0.05	0.025

Figure 5. **(D) Total colocalization area**

Group	Summary	*p* value	
CTL vs. PPA	***	<0.001	0.0002
CTL vs. PPA + SER	ns	ns	0.3127
PPA vs. PPA + SER	**	<0.01	0.0027

(D) Average colocalization size

Group	Summary	*p* value	
CTL vs. PPA	**	<0.01	0.0026
CTL vs. PPA + SER	ns	ns	0.9927
PPA vs. PPA + SER	**	<0.01	0.0031

Figure 6.

(B) Lamp1 integrated density

Group	Summary	*p* value	
CTL vs. PPA	ns	ns	0.162150718
CTL vs. PPA + SER	****	<0.0001	2.0057E-06
PPA vs. PPA + SER	**	<0.01	0.002658303

(B) CTSD integrated density

Group	Summary	*p* value	
CTL vs. PPA	ns	ns	0.198
CTL vs. PPA + SER	ns	ns	0.3681
PPA vs. PPA + SER	*	<0.05	0.0115

(D) Prepro CTSD

Group	Summary	*p* value	
CTL vs. PPA	*	<0.05	0.01684855
CTL vs. PPA+SER_100uM	ns	ns	0.220110645
CTL vs. PPA+SER_1mM	ns	ns	0.742786083
PPA vs. PPA+SER_100uM	ns	ns	0.318346241
PPA vs. PPA+SER_1mM	**	<0.01	0.004557476
PPA+SER_100uM vs. PPA+SER_1mM	ns	ns	0.053120381

(D) Mature CTSD

Group	Summary	*p* value	
CTL vs. PPA	*	<0.05	0.01176152
CTL vs. PPA+SER_100uM	ns	ns	0.068776054
CTL vs. PPA+SER_1mM	ns	ns	0.987873595
PPA vs. PPA+SER_100uM	ns	ns	0.595546612
PPA vs. PPA+SER_1mM	*	<0.05	0.017984725
PPA+SER_100uM vs. PPA+SER_1mM	ns	ns	0.10793986

(D) Lamp1

Group	Summary	*p* value	
CTL vs. PPA	ns	ns	0.130169388
CTL vs. PPA+SER_100uM	***	<0.001	0.000383434
CTL vs. PPA+SER_1mM	ns	ns	0.216728239
PPA vs. PPA+SER_100uM	ns	ns	0.129359757
PPA vs. PPA+SER_1mM	ns	ns	0.441288019
PPA+SER_100uM vs. PPA+SER_1mM	ns	ns	0.05003386
